# Müller glia derived EVs promote neurite recovery of an enriched population of retinal ganglion like cells derived from hESC retinal organoids after damage

**DOI:** 10.1038/s41598-026-42089-8

**Published:** 2026-03-03

**Authors:** Karen Eastlake, William D. B. Lamb, Dhani Tracey-White, Keiichi Shibagaki, Peng T. Khaw, Hari Jayaram, G. Astrid Limb

**Affiliations:** 1 National Institute for Health and Care Research (NIHR) Biomedical Research Centre at Moorfields Eye Hospital NHS Foundation Trust and UCL Institute of Ophthalmology, 11-43 Bath Street, EC1V 9EL London, UK; 2 Discovery Research, Ophthalmology Innovation Center, Pipeline Creation, Santen Pharmaceutical Co. Ltd., Osaka, Japan

**Keywords:** Muller glia, Retina, Stem cells, Retinal organoids, Retinal ganglion cells, Degeneration, Neuroprotection, Cell biology, Neurology, Neuroscience

## Abstract

Membrane-bound extracellular vesicles (EVs) released by Müller glia contain microRNA (miRNA) and proteins with the potential to be beneficial in providing neuroprotection in retinal degenerative conditions. The aim of this study was to examine the neuroprotective effect of Müller glia derived EVs in human ESC-derived neuronal cells containing RGCs. Cells were isolated from 40–50-day-old retinal organoids and cytotoxicity was induced by addition of NMDA (1mM) for 24 h, followed by treatment with a population of Müller cell derived EVs for a further 24 h. Isolated RGC-enriched cultures expressed characteristic RGC markers such as βIII tubulin, Brn3b, RBPMS, γ-synuclein, Thy1 and NMDAR1. Exposure of this cell population to NMDA lead to a significant reduction in the average neurite length which then recovered with exposure to Müller cell-derived EVs. Investigation of kinase activity revealed increased apoptotic signalling via activation of P38 and p53 after NMDA addition to RGC-like cultures and pro-survival signalling by activation of RSK1/2/3 after addition of EV to the NMDA-damaged cells, suggesting EVs derived from Müller glia support the survival of the RGC-enriched cultures. It is hoped that these observations aid future investigations to assess new EV-related therapies to treat neuronal injury occurring in retinal degenerative conditions such as glaucoma.

**Supplementary Information:**

The online version contains supplementary material available at 10.1038/s41598-026-42089-8.

## Introduction

Müller glia, the main glial cell in the retina, derive from the same progenitor pool as the neural retina and play key roles in providing homeostatic, metabolic and structural support to all retinal neurons^1^. Müller glia have the unique property of being able to regenerate the neural retina in species such as the zebrafish, after injury^2^. Although regeneration is not observed in human retina, we have identified stem cell properties in these cells where they can be induced to become precursors of retinal ganglion cells (RGCs) or photoreceptors^3–5^. In addition, transplantation of human Müller glia derived from either adult human retina^6^ or retinal organoids derived from pluripotent stem cells has shown partial improvement in visual function when transplanted to rodent models of RGC depletion^7^. However, in these studies, there was no integration of the transplanted cells into the host retina, suggesting that Muller cells are releasing neuroprotective factors within the local retinal environment.

Extracellular vesicles (EVs), produced by almost all cells, are lipid-bound nanocarriers released either through blebbing of the external cellular membrane (microvesicles), or through inward budding of the endosomal membrane, which produces vesicle filled compartments (multi-vesiculated bodies - MVB)^8^. MVBs that fuse with the cell membrane are then released to the extracellular space (commonly known as exosomes) to perform functions in intercellular communication, delivering biomolecules such as miRNAs, protein and lipids that can affect downstream signalling in recipient cells^8^. EVs are attracting growing attention as a therapeutic avenue in regenerative medicine due to their biological activity, availability, cell-based origin and low immunogenicity. Multiple cell sources of EVs have been investigated with regards to retinal degeneration and have shown some therapeutic benefit in animal models. Mesenchymal stem cells are currently a popular source of EV and have shown therapeutic benefit in rodent models of retinal detachment by reducing inflammation and supressing apoptosis^9^, and display neuroprotective effects on RGCs using an optic nerve crush model^10^. The therapeutic effect of these EV have been attributed to multiple miRNAs including MiR-21, MiR-126, and MiR-146a^10^. More recently, studies have investigated the use of EVs derived from retinal pigment epithelial cells (RPE). In a mouse model of retinal photoreceptor degeneration induced by N-methyl-N-nitrosourea (MNU), subretinal delivery of RPE-derived EVs were shown to reduce photoreceptor death by reducing inflammation and oxidative damage^11^, although the exact method has not been defined. It is reasonable therefore, to suggest that some of the neuroprotective ability of human Müller glia in animal models of retinal degeneration may be ascribed to the release and content of EVs. We have recently demonstrated that membrane-bound extracellular vesicles (EVs) released by Müller glia contain microRNA (miRNA) and proteins that may regulate anti-apoptotic processes and therefore be beneficial in providing neuroprotection in retinal degenerative conditions^12^.

Several studies have used rodent models that may include in vivo animal studies, ex vivo retinal tissues or isolated primary RGC cultures to investigate mechanisms of RGC biology and neuroprotection. Although they have aided in the understanding of RGC neuroprotection, advances in the development of 3D retinal organoid formation from human stem cells has provided a novel method with which to isolate and culture RGCs that are affected in glaucoma. It has been shown that these cells can be directly isolated or differentiated from human embryonic (ESC) or induced pluripotent stem cells (iPSC)^13,14^ and used as an in vitro model to investigate neuroprotective mechanisms^15,16^ aiding in the development of practical in vitro models that resemble human disease whilst reducing the need for animal tissues.

In the current study we have isolated and characterised a population of RGC-enriched cultures from human stem cell retinal organoids. In addition, we have shown that Müller glia derived EVs can lead to RGC repair as demonstrated by increases in their average neurite length after NMDA-induced damage, that was associated with increased activation of RSK1/2/3 after EV treatment. This suggests that EVs may be supporting the survival and neuroprotection of neuronal and RGC-like cells and warrants further investigations.

## Results

### Müller glia extracellular vesicles

**Fig. 1:**
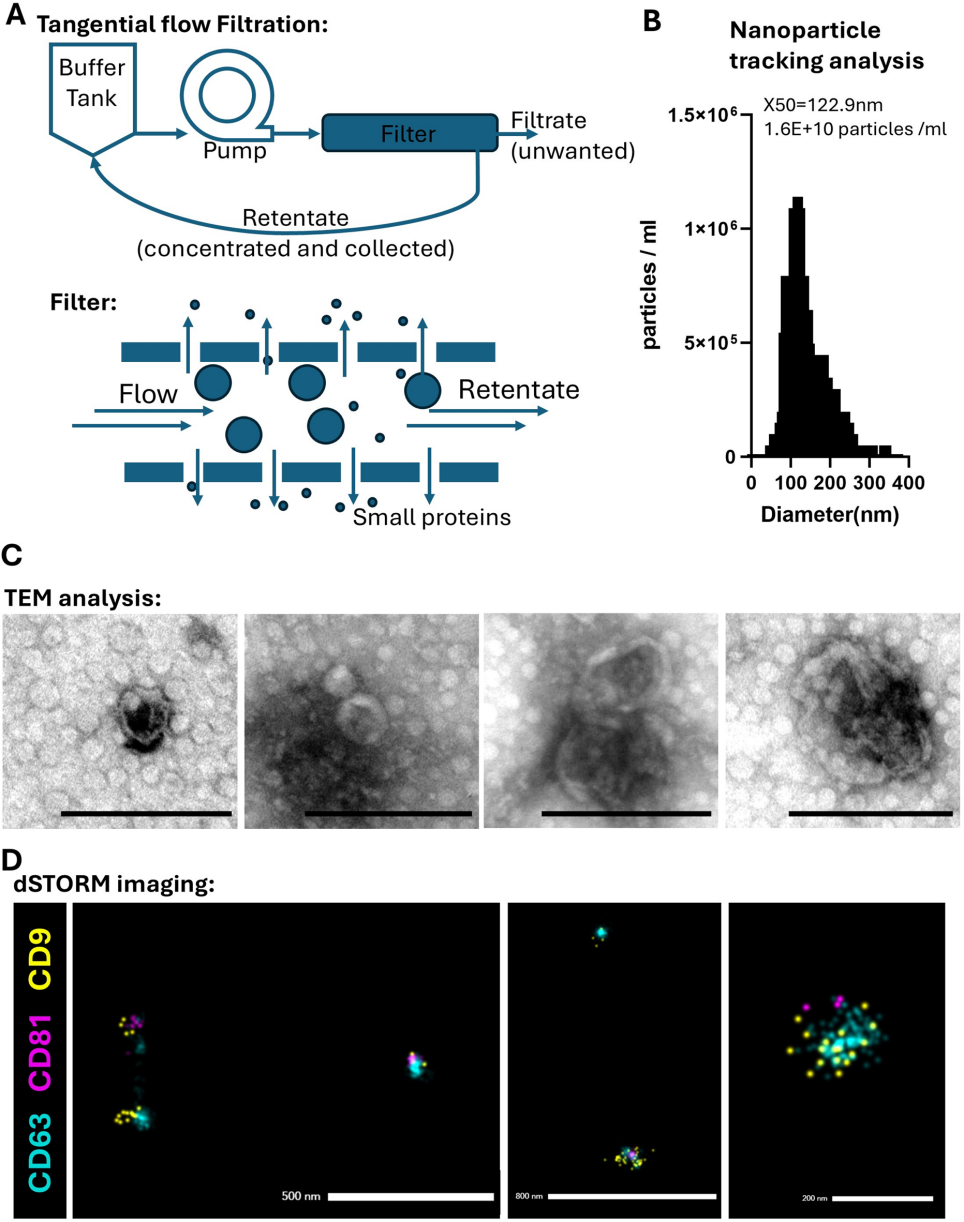
Characterisation of Müller glia derived extracellular vesicles. (**A**) Illustrative diagram shows the method of tangential flow filtration, used to purify extracellular vesicles from collected tissue culture medium. (**B**) Size distribution and concentration of EV were determined by NTA. (**C**) TEM images show individual EV with double membrane highlighted by arrows (Scale bars 200 nm). (**D**) EVs labelled with antibodies to CD9 (yellow), CD81 (magenta) and CD63 (Blue) were examined by dSTORM imaging (scale bars in panels left to right, 500 nm, 800 nm, 200 nm).

Extracellular vesicles (EV) were isolated from a population of hESC retinal organoid derived Müller glia cells by tangential flow filtration using a 300 kDa filter to enrich for small EV and to remove small impurities such as proteins (Fig. 1A). Nanoparticle tracking analysis was conducted to estimate the particle concentrations and size distribution of the Müller EV. The median particle size (X50) was determined as 122.9 nm, with a concentration of 1.6e10 particles/ml (Fig. 1B). Transmission electron microscopy analysis of the EV confirmed the presence of a characteristic double membrane consistent with previous studies^12,17^ and showed that size distribution aligned with the NTA (Fig. 1C, scale bar 200 nm). Super resolution imaging was conducted to confirm EV identity by detection of three typical EV transmembrane proteins, CD9, CD81 and CD63 (Fig. 1D). Representative images of individual EV particles show positive expression for all three tetraspanins, each dot representing a single transmembrane protein (Fig. 1D, EV shown at different magnifications).

### Retinal organoid development and isolation of RGC-enriched cultures

**Fig. 2:**
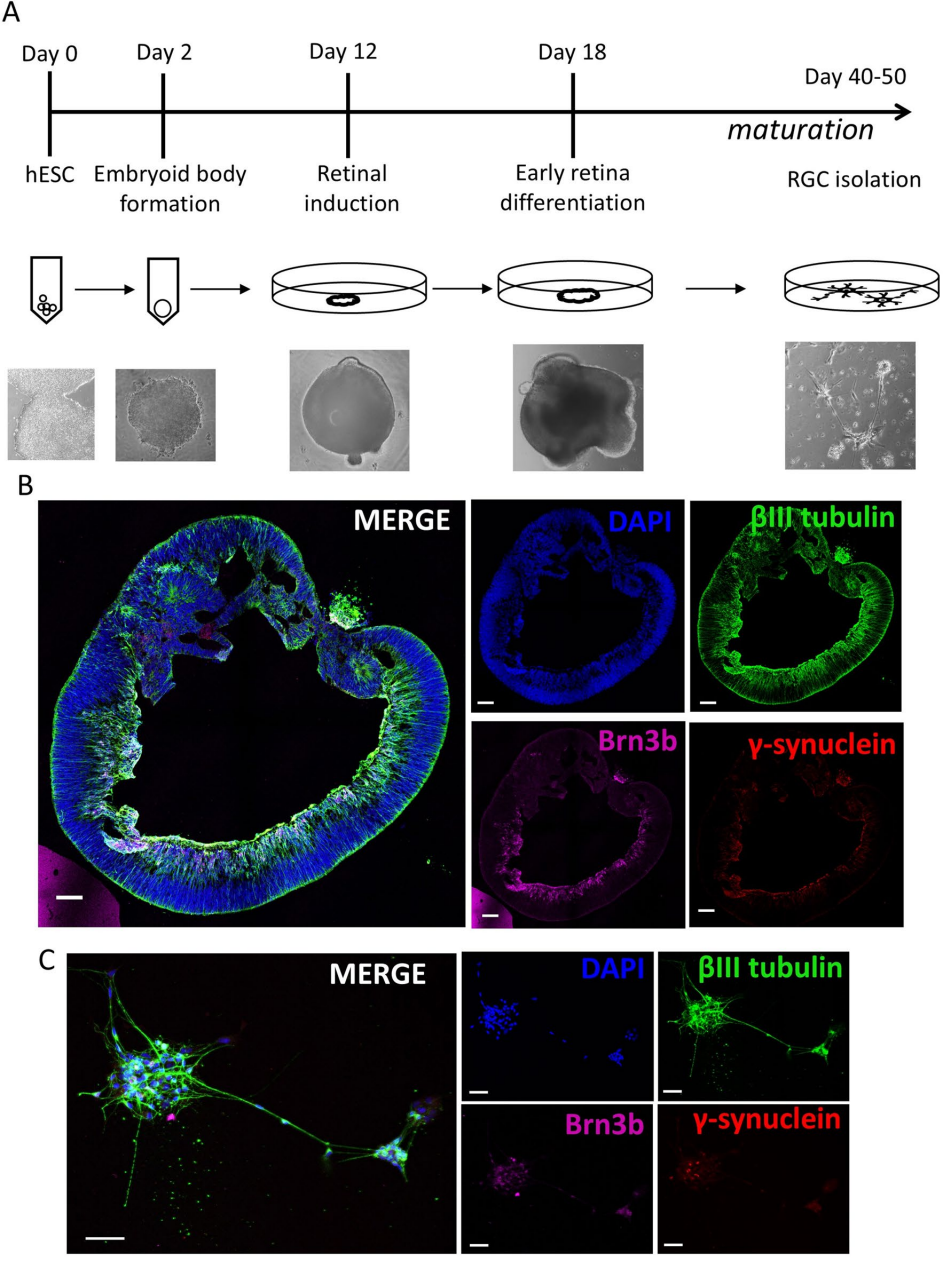
Human ESC-Retinal organoid differentiation and RGC isolation. (**A**) Timeline of differentiation of human ESCs into retinal organoids and isolation of RGC-enriched cultures. (**B**/**C**) Representative fluorescent confocal images of (**B**) day 45 retinal organoids (Scale 100 μm) and (**C**) isolated RGC-enriched neuronal cultures showing positive staining of Brn3b (magenta), γ-synuclein (red), and βIII tubulin (green). (Scale 50 μm) Nuclei counterstained with DAPI (blue).

Human embryonic stem cells were differentiated using a previously published protocol^7,18^ towards early retinal tissue identity from which retinal ganglion (RG)-like cells were isolated (Fig. 2A). Development of cells expressing typical RGC markers was confirmed by immunofluorescence staining showing positive expression of βIII tubulin, BRN3B and gamma synuclein in the inner cell layers of retinal organoids from 20 days onwards after initiation of differentiation (Fig. 2B). Organoids aged 40–50 days were used to isolate neural and RGC-enriched cultures by single cell dissociation and seeding onto laminin-coated flasks in the presence of a specific neural medium to encourage neural cell growth. Cell cultures consisted of small cell clusters with multiple neurite processes as shown by βIII tubulin expression. A proportion of these neural cell clusters also expressed the RGC associated markers BRN3B and gamma synuclein (Fig. 2C).

### Isolated neural clusters show positive expression for RGC markers

**Fig. 3:**
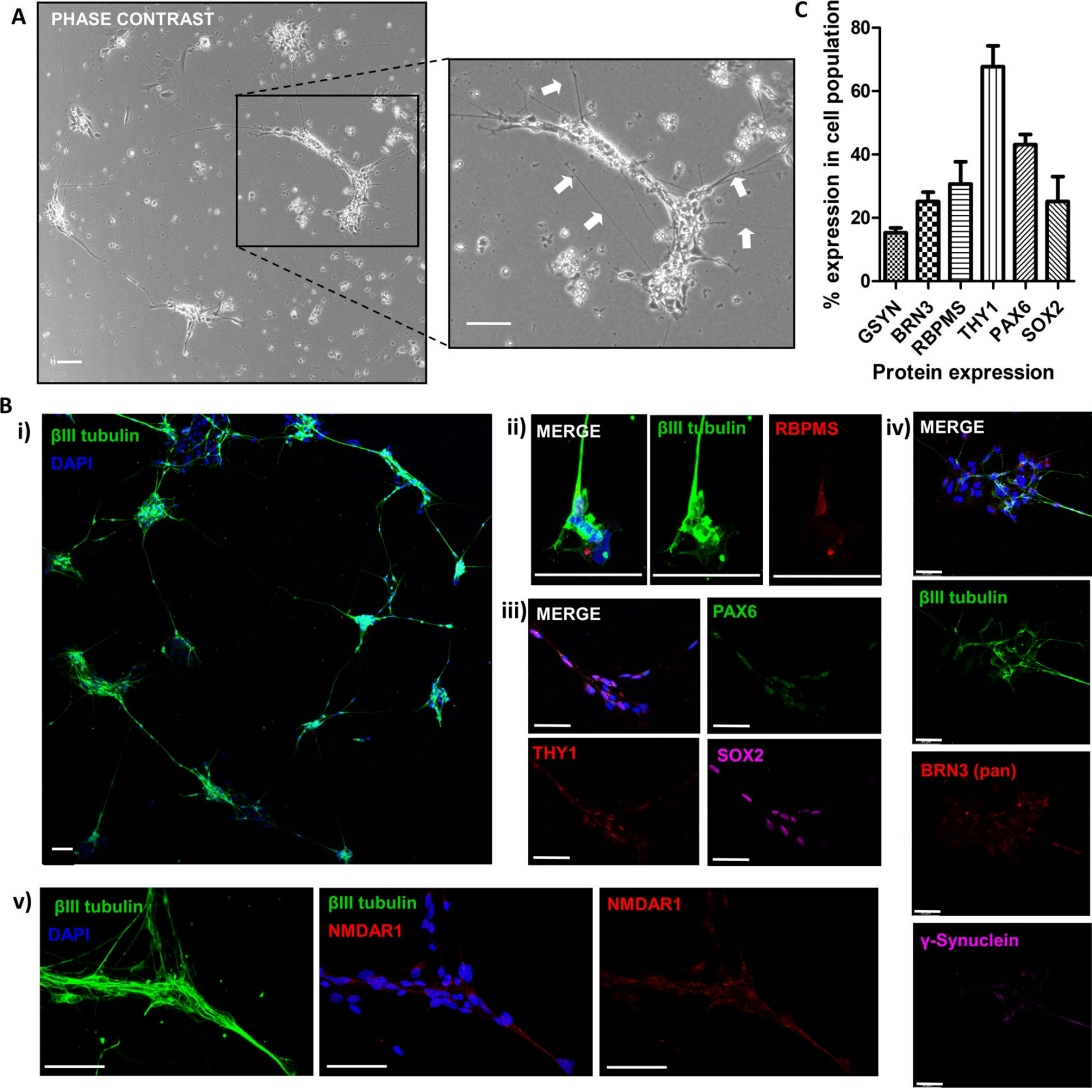
Characterisation of hESC-retinal organoid derived RGC-like cultures. (**A**) Representative phase contrast image of RGC-enriched neuronal clusters (**B**) Representative fluorescent confocal images of RGC-enriched neuronal clusters showing low power magnification of (**i**) βIII-tubulin (green) positive neuronal clusters, and higher magnification of (**ii**) βIII-tubulin (green) and RBPMS (red) positive cells; (**iii**) PAX6 (green), THY1 (red), and SOX2 (magenta) positive cells; (**iv**) βIII-tubulin (green), BRN3 (pan, red), and γ-synuclein (magenta) positive cells; and (**v**) βIII-tubulin (green), and NMDAR1 (red). Scale = 50 μm. Nuclei counterstained with DAPI. (**C**) Bar graphs show percentage expression of the RGC markers in the isolated cell population (mean ± SEM).

Further characterisation of these neural cell clusters was performed by immunofluorescence analysis after isolation from retinal organoids grown on laminin-coated flasks. Phase contrast microscopy images showed small cell clusters or dissociated cells extending out neural-like processes (arrows) (Fig. 3A). Confocal microscopy images of cell preparations immunostained for βIII tubulin showed that neural extensions from cell clusters were also positive for this neuronal marker (Fig. 3Bi). Expression of typical markers associated with RGCs were also examined in the isolated cells. The RNA binding protein with multiple splicing (RBPMS) which is uniquely expressed in mature RGCs in the retina, was expressed in a small proportion of the isolated cells as shown by fluorescence staining (Fig. 3Bii). Cells in isolated clusters were also shown to express paired box protein 6 (PAX6), which is involved in RGC fate determination, the neural progenitor marker SRY (sex determining region Y)-box 2 (SOX2) and the cell surface protein cluster of differentiation 90 (CD90 or THY1), which is highly expressed in RGCs, but may also be found expressed by retinal precursor cells and fibroblast-like cells^19^ (Fig. 3Biii). A proportion of cells also express BRN3, an important factor in RGC development, and gamma synuclein, which is normally highly expressed by adult RGCs (Fig. 3Biv). The presence of NMDA receptor 1 (NMDAR1) on a high proportion of isolated cells was also confirmed (Fig. 3Bv). Quantification of isolated neural cell populations expressing RGC markers showed that above 65% of isolated cells were positive for THY1 and over 40% for PAX6 (Fig. 3C). RBPMS and BRN3 expression was observed in between 20 and 40% of isolated cells, whereas the more mature RGC marker gamma synuclein was only expressed in approximately 15% of the isolated cells (Fig. 3C). This data indicates that the cell preparation obtained is not a pure RGC-like population, with RGC likely comprising 30–40% of our isolated population. Whilst it is unlikely that any photoreceptor type cells are present due to the early ages of the organoids used, it is possible that other neural cells may be present such as neural progenitor type cells, and even though rare in organoids, amacrine or horizontal cells.

### EVs promote neurite outgrowth in RGC-enriched cultures

**Fig. 4:**
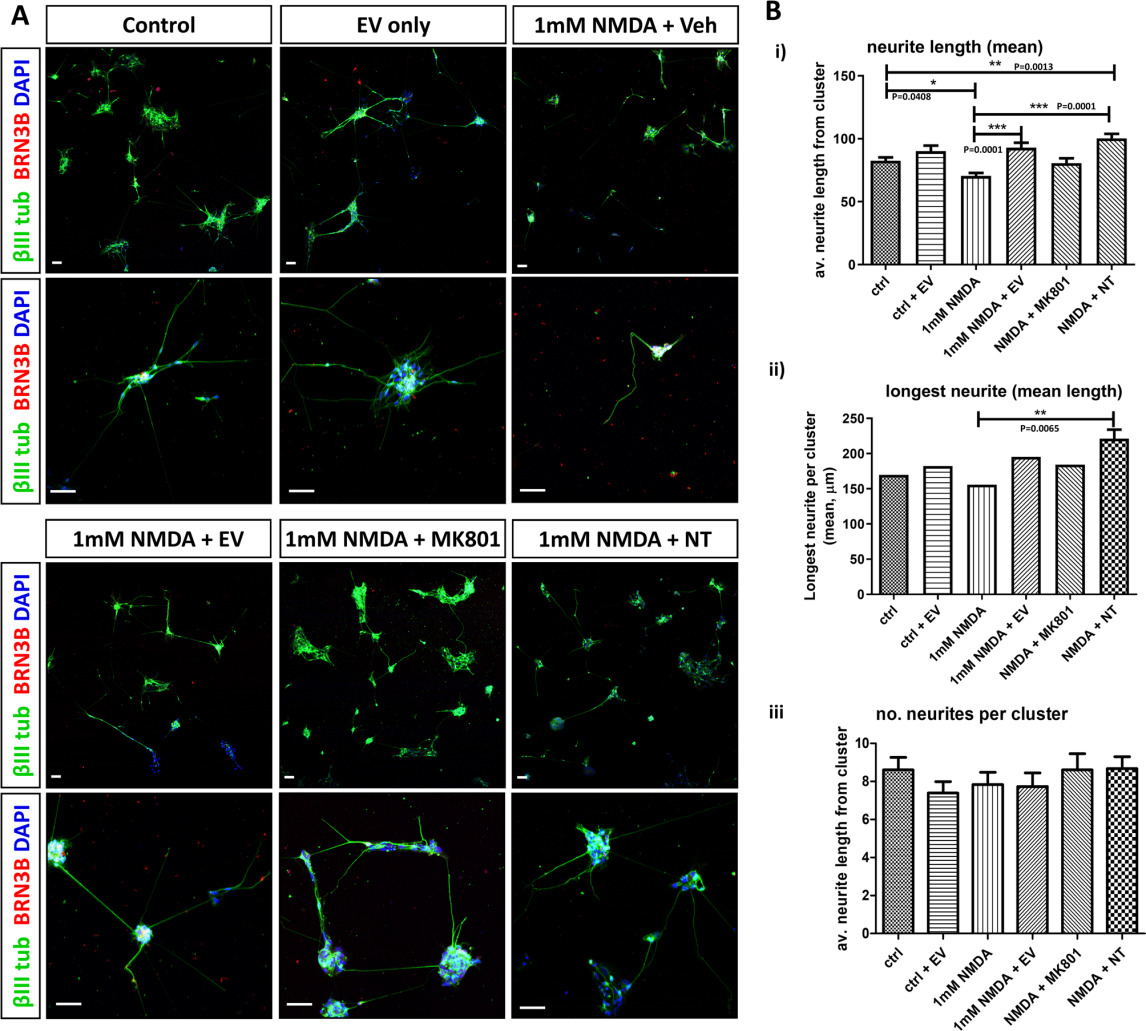
Müller derived EVs promote neural outgrowth of human RGC-like cells. (**A**) Representative fluorescent confocal images show clusters of RGC-enriched cultures treated with cell culture medium only (control), EV only, 1mM NMDA + vehicle, 1mM NMDA + EV, 1mM NMDA + MK801 or 1mM NMDA + NT stained with antibodies to βIII-tubulin (green) and BRN3 (red) and counterstained with DAPI (blue). All scale bars indicate 50 μm (**B**) Bar charts show assessment of cell treatments on (**i**) mean neurite length from each cluster, (**ii**) longest neurite (mean) from each cluster and (**iii**) number of neurites per cluster. One-way ANOVA with Bonferroni correction. *P < 0.05; **P < 0.01; ***P < 0.001. *n* = 4 (mean ± SEM.).

To assess the neuroprotective ability of EVs, we compared the morphological differences in the RGC-enriched cultures after a 24 h NMDA induced damage followed by culture with or without EVs isolated from Müller glia. Although the uneven distribution of the isolated cells and clusters made interpretations and analysis challenging, careful examination of low and high power images of cell clusters and neurites (shown in Fig. 4A) made analysis possible. All RGC-enriched clusters analysed contained cells positive for BRN3B and βIII tubulin. As indicated by βIII tubulin staining (Fig. 4A), when 1mM NMDA alone was added to the cultures, neurite extensions were typically observed shorter and clusters were sparse in comparison to NMDA damaged cells cultured with either EVs, a neurotrophin (NT) cocktail or the NMDA receptor inhibitor MK801. Furthermore, image J analysis of the cell preparations showed a significant decrease in the average neurite length from cell clusters exposed to 1mM NMDA for 24 h as compared to control cells (Fig. 4Bi ; *p* = 0.0408). A small increase in the average neurite length was noted with the addition of EV to control cells after 24 h, however this was not statistically significant (Fig. 4Bi). Cells exposed to NMDA followed by 24 h culture with Müller glia derived EVs or the NT cocktail showed a significant increase in the average neurite lengths growing outwards from the clusters (Fig. 4Bi; *p* < 0.0001 for both) As expected, the average neurite length of cells treated with NMDA plus the NMDAR antagonist MK-801, showed no differences as compared to control cells (Fig. 4Bi). Image J measurements of the longest neurite from each cell cluster showed a similar trend to the average neurite length analysis, however the longest neurite measurement was only statistically significant for the NMDA + NT treatment as compared to 1mM NMDA alone (Fig. 4Bii; *p* = 0.0065). We did not observe any significant difference in the number of neurites per cluster (Fig. 4Biii), suggesting that EVs enhance neurite length with little influence on neurite number.

### Confirmation of EV uptake

**Fig. 5:**
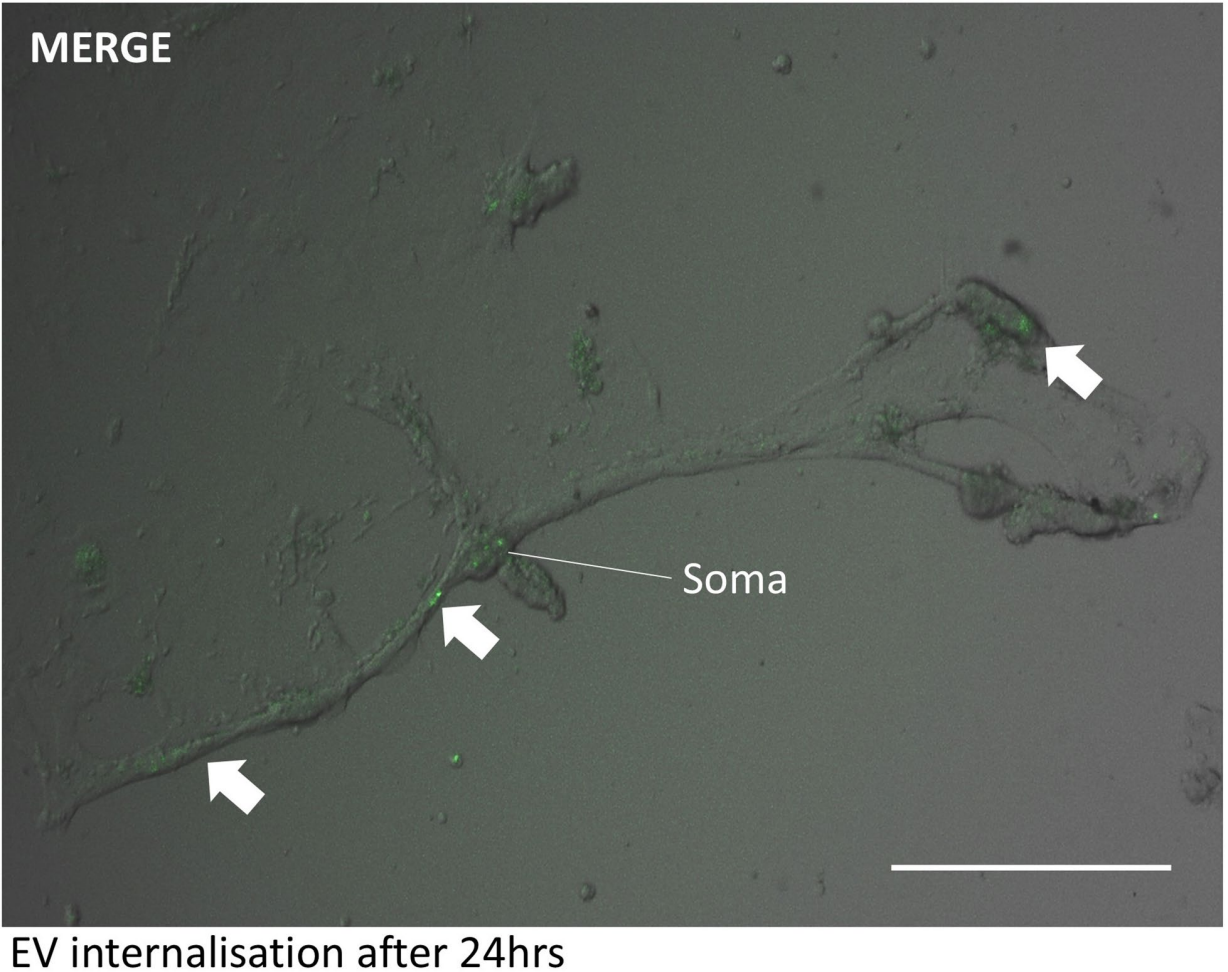
Müller glia EV uptake by RGC-enriched cultures. Representative phase contrast and fluorescent image show internalisation of labelled EV clusters after 24 h. EVs labelled with ExoGlow protein labelling kit GFP (green). White arrows show internalisation of EV clusters to neural extensions and cell soma (scale 100 μm.)

Uptake of Müller derived EVs to RGC-enriched cultures was confirmed by the confocal imaging of cells treated with fluorescently labelled EVs. As seen in Fig. 5, the representative qualitative image shows EVs accumulated around the soma and neurites after 24 h incubation (white arrows - Fig. 5).

### Examination of potential signalling targets of Müller derived EVs

**Fig. 6:**
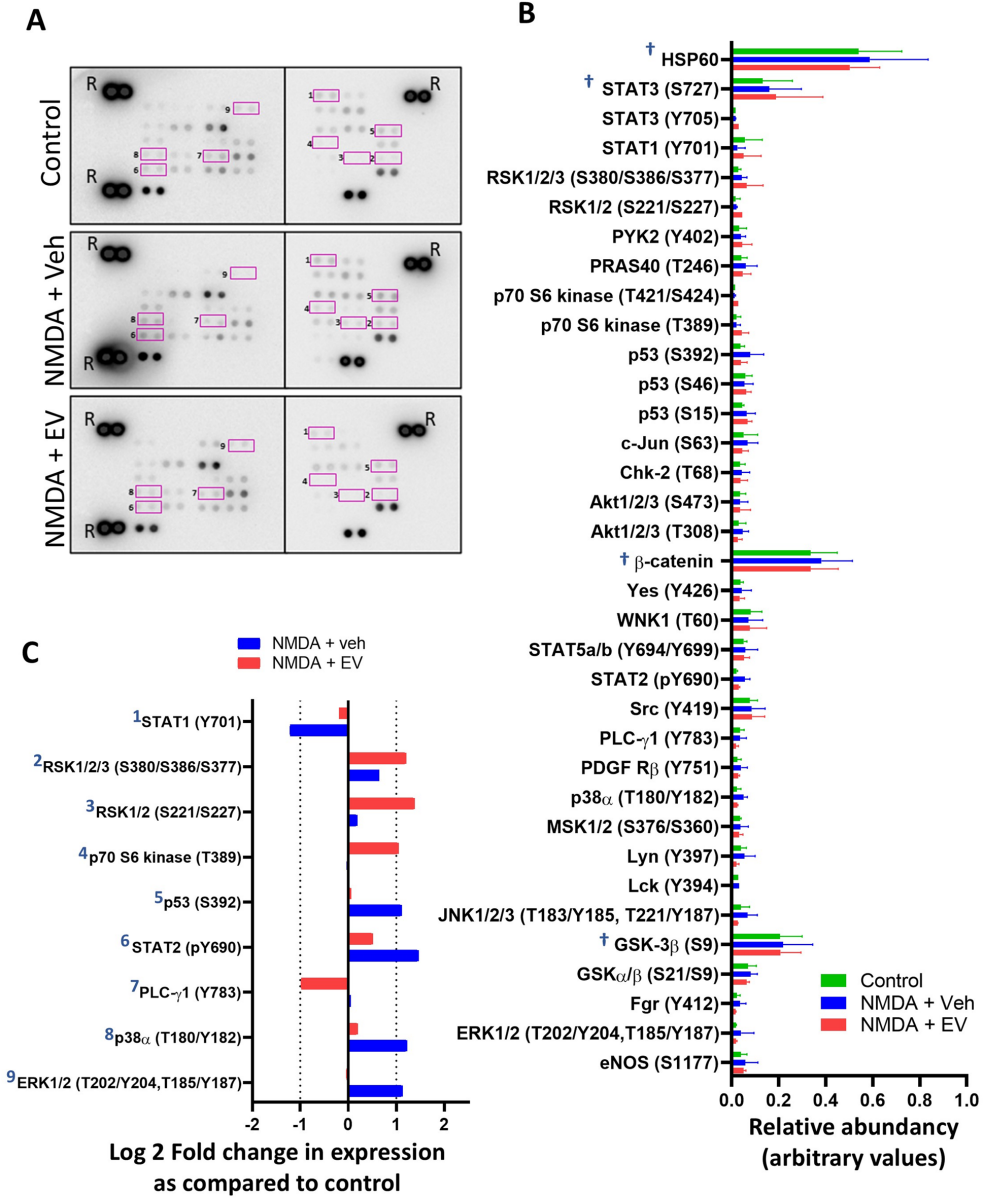
Effect of NMDA and EV treatment on the phosphorylation of protein kinases after 24 h. Proteome Profiler Human Phospho-Kinase Array analysis of untreated control RGC-enriched cultures (control), as compared to those treated with NMDA for 24 h with or without Müller EV treatment for a further 24 h (NMDA + vehicle, NMDA + EV). (**A**) Representative chemiluminescent images show the relative expression of each phosphorylated protein. R= reference spot used for normalisation. (Full dot blot chemiluminescent images can be found in supplementary Fig. 2). Those numbered 1–9 are highlighted in graph C. (**B**) Histogram shows the relative abundance of each of the detected phospho-kinases as judged by Image J. *n* = 4 (mean ± SEM) .(**C**) Histogram show kinases that showed averaged log2 fold change (log2 FC) in expression with a cutoff of +/- 1 log2 FC in NMDA + vehicle or NMDA + EV as compared to expression by control retinal ganglion cells. † highlights the proteins with highest levels of detected phosphorylation.

We investigated the potential downstream targets of our Müller EV preparations in the RGC-enriched cultures by a membrane based sandwich immunoassay to detect the relative phosphorylation levels of multiple kinase phosphorylation sites after 24 h. This assay showed that overall several phospho-proteins were detected at high levels in the RGC-enriched neuronal cultures. These included HSP60, STAT3 (S727), β-catenin, and GSK-3β (S9) which showed no differences between the different culture treatments (NMDA + Veh, NMDA + EV or control; marked with a †) (Fig. 6A/B). We identified, showed marked changes in phosphorylation levels when comparing treatment conditions (NMDA + vehicle or NMDA + EV) to the control cells. Increased phosphorylation was detected in ERK1/2 (T202/Y204, T185/Y187), when cells were incubated with NMDA + vehicle as compared to controls or NMDA + EV-treated cells. A similar pattern was observed with P38a (MAPK, T180/182), STAT2 (pY690) and P53 (s392). Phosphorylation levels of PLC gamma1 (Y783) expression did not alter from control after addition of NMDA+ vehicle to the RGC-like cells, however with NMDA + EV treatment, there was a marked downregulation after 24 h. Increased phosphorylation of RSK1/2 (S221/S227) and RSK1/2/3 (S380/S386/S377) was observed in cells treated with NMDA + EV as compared to control. Lastly, STAT1 (Y701) expression showed a marked fold-downregulation of phosphorylation after NMDA + vehicle treatment in the cells as compared to control, which was not observed when cells were treated with NMDA + EV (Fig. 6C).

## Methods

### Retinal organoid formation and isolation of Müller glia

Human embryonic stem cells (RC9 cells Research grade, Roslin cells/ UK stem cell bank or Man15 cells Research grade, UMAN, UK stem cell bank) were maintained in Stem-Pro complete medium (A1000701; Thermo Fisher Scientific, UK) containing 1% ×100 penicillin/streptomycin and grown on 0.05 mg/ml human Vitronectin (A14700; Thermo Fisher Scientific) coated plates. Retinal organoid differentiation was induced using previously published protocols^7,18^. Briefly, cells were seeded to a density of 9,000 cells per well in a v-bottomed 96‐well plate (PrimeSurface Sumilon low adhesion, Alpha Laboratories, U.K.) in Glasgow Minimum Essential Medium (GMEM) with l‐glutamine containing 20% KOSR (ThermoFisher), 1% ×100 sodium pyruvate (ThermoFisher, U.K.), 1% ×100 nonessential amino acids (ThermoFisher), 1% ×100 penicillin/streptomycin (ThermoFisher, U.K.) and 50µM β‐mercaptoethanol, containing 20µM ROCKi and 3µM Wnt antagonist (Millipore, Watford, U.K.). On days 2, 5 and 9, wells were fed with 100 µl of the same media supplemented with 1% Matrigel. On days 12 and 15, embryoid bodies (EBs) were incubated in the above media (without ROCKi and WNT antagonist) with the addition of 10% FBS, 1% Matrigel and 100nM smoothened agonist (SAG; Millipore, Watford, U.K.). On day 18, medium was replaced with Dulbecco’s modified Eagle’s medium/F12‐glutamax (DMEM-F12) containing 10% FCS, 1% ×100 N2 supplement (ThermoFisher, U.K.; 1% penicillin/ streptomycin/ amphotericin and 0.5µM retinoic acid (Sigma‐Aldrich, U.K.). Retinal organoids were then fed twice weekly with the above media containing N2 supplement and retinoic acid. Between days 30 to 50, neural retinal tissue was dissected and isolated from the remaining embryoid body to mature in culture. Müller glia cells were isolated from retinal organoids (70 + day-old) by dissociation into single cells using a gentle cell dissociation reagent (Stem Cell Technologies, UK) (5 min at 37 °C), and plated onto fibronectin coated tissue culture plates (50 µg/ml) in the presence of fibroblast growth factor (FGF) (20 ng/ml) and epidermal growth factor (EGF) (20 ng/ml) in DMEM containing 10%FCS and 1% penicillin–streptomycin. These cells have been fully characterised previously^7,18^. Müller glia cell cultures were maintained in this culture medium for up to 10 passages to isolate EVs. All cells and organoids were maintained at 37 °C, 5% CO2, and atmospheric O2.

### EV isolation

Extracellular vesicles were purified from Müller glia cell culture supernatants using ultracentrifugation and tangential flow filtration based upon previously published protocols^12^. Briefly, Müller cell cultures were grown as described above and then washed three times in particle-depleted PBS. Cells were then maintained in vesicle-depleted media for 24 h, which was then collected, and centrifuged at 300 x g for 10 min. The cell pellet was discarded and the supernatant collected for further filtration by tangential flow (Minimate™ EVO TFF System, Pall, Cytiva Life Sciences). EVs were then pelleted by centrifugation at 100, 000 × g for 120 min at 4 °C. The pellet was finally resuspended in 100 µL of sterile, particle-depleted PBS. The concentration and size profile of EV preparations were quantified by nanoparticle tracking analysis (NTA). Measurements were made using a NanoSight LM10 instrument equipped with a 405 nm LM12 module and EM-CCD camera (NanoSight, Malvern Panalytical Ltd, Malvern, UK).

### TEM

EVs were fixed in 1/2 Karnovsky’s solution (2.5% glutaraldehyde, 1% paraformaldehyde buffered to pH 7.4 with 0.08 M sodium cacodylate-HCl buffer)(Agar Scientific Ltd). Droplets of EV suspensions were then adsorbed onto 1% formvar coated grids for 10 min (copper grids and formvar powder from Agar Scientific Ltd). Negative staining was then performed by passing the grids through 2 successive washes of double distilled water followed by a 10 min incubation in a 2% uranyl acetate solution (w/v) (Agar Scientific Ltd), after which excess stain was blotted on Whatman and grids air-dried. Imaging was conducted on a JEOL JEM1400plus transmission electron microscope operating at 80 kV (JEOL UK) with image capture by a Deben NanoSprint12 camera using AMT software.

### Super resolution imaging

EVs were characterised for the presence of CD9, CD81 and CD63 by using the EV profiler kit (900 − 00242, ONI UK) and imaged using direct stochastic optical reconstruction microscopy (dSTORM). dSTORM was conducted using a Nanoimager S Mark II microscope from ONI (ONI, Oxford, UK) equipped with a 100×, 1.4NA oil immersion objective, an XYZ closed-loop piezo 736 stage, and dual emission channels split at 640 nm. Briefly, approximately 10ul of EV solution (1 × 10^9^EV/ml) was added to the microfluidic chip and left to incubate for 75 min before adding fixative supplied by the kit. Detection antibodies (anti-CD81 -647; anti-CD63-561; anti-CD9-488) were then incubated for a further 50 min. Chips were then washed, ready for imaging. Freshly prepared dSTORM imaging buffer (supplied) was added immediately prior to image acquisition. The acquired images were processed using ONI’s online analysis platform (CODI).

### Isolation of RGC-enriched cultures from retinal organoids

Retinal organoids were differentiated using the methods described above. To isolate retinal ganglion like cells, organoids aged between 40 and 50 days after initiation of differentiation were used, when they expressed the peak number of RGC cells. A pool of 15–25 organoids were used for each isolation depending on the number of cells required and incubated in gentle cell dissociation reagent (Stem Cell Technologies, UK) (10 min at 37 °C), with regular agitation using a P1000 to break up organoid clumps. Cells were collected in RGC-neural media (RGC-NIM) which consisted of DMEM-F12 with addition of 1% N2 supplement, 1% NEAA, 0.1% heparin and 1% penicillin–streptomycin, and centrifuged at 1000 x g for 5 min. Cells were resuspended in RGC-NIM media and cultured on laminin (20 µg/ml) coated 24 well plates to a density of 60,000 cells/ well. RGC-like cells were characterised by immunofluorescence staining for RGC marker expression. Four random visual fields were used for cell analysis and counts.

### Induction of neuronal cell damage and EV treatment

Isolated RGC-enriched cells were cultured in the presence of NMDA (1mM) in RGC-NM for 24 h. RGC cells were then washed three times in DMEM F12 prior to addition of approximately 10^9^ EV for a further 24 h. A vehicle consisting of PBS was used for negative controls, a neurotrophin cocktail (BDNF, CNTF, forskolin) was used for a positive control and a noncompetitive NMDA receptor antagonist MK-801 was used as an additional control. At least three replicates were performed consisting of independent differentiations and isolations of RGC-enriched cultures which were conducted on different days.

### Immunofluorescence staining

Cells were grown on to laminin (20 µg/ml) coated Ø13mm glass coverslips placed in 24 well plates. After experimental conditions were applied, cells were fixed in 4% paraformaldehyde in phosphate buffered saline (PBS) for 10 min at room temperature. Cells were then blocked for 1 h in tris buffered saline (TBS) + 0.3% triton + 5% donkey serum before the addition of the primary antibody (diluted in blocking buffer). Primary antibodies (see Supplementary Table 1) were added and plates incubated overnight at 4 °C, followed by three washes with TBS for 5 min each. After addition of secondary antibodies (Alexa flour, Invitrogen, U.K. 1:500 in TBS + 0.3% triton), plates were incubated for 3 h at room temperature in the dark. Cells were then washed in TBS and coverslips mounted with Fluoroshield Mounting Medium containing 4′,6-diamidino‐2‐phenylindole (DAPI; Abcam, Cambridge, U.K.) onto microscopy slides (face down) and edges sealed with nail varnish. Secondary only negative controls were performed, and showed no unspecific reactivity in the isolated cells (See supplementary Fig. 1).

### Neurite measurements

Image J was used to manually measure neurite length and numbers, cell cluster areas and to perform cell counts. At least 4 randomly selected visual fields (each containing at least two cell clusters) were analysed per replicate (See supplementary Table 2). One way ANOVA with Tukey multiple comparisons test was used to calculate significance.

### Proteome profiler human phospho-kinase array

The Proteome Profiler Human Phospho-Kinase Array (ARY003C; R&D Systems, UK), was used to determine the relative levels of phosphorylation of a panel of 37 kinase phosphorylation sites. The assay was carried out according to the manufacturers instructions. To prepare the samples, cells were grown on to laminin (20 µg/ml) coated 6 well plates. After experimental conditions were applied protein was isolated using RIPA buffer (R0278; Sigma-Aldrich, UK) containing 0.6 M sodium orthovanadate (S6508, Sigma-Aldrich, UK), 0.5 M DTT ( D0632; Sigma-Aldrich, UK), protease inhibitor cocktail (P8340; Sigma-Aldrich, UK) and 0.5 M PMSF (P7626, Sigma-Aldrich, UK). Protein was measured using the BCA kit (23225, Thermo Fischer, UK) and 50ug protein was used for each membrane of the assay. The assay was carried out according to manufacturers instructions (ARY003C; R&D Systems, UK). The array was replicated four times. Full dot blot chemiluminescent images can be found in supplementary Fig. 2 and calculated normalised arbitrary vales can be found in Supplementary Table 3.

## Discussion

In this study we have defined a simple in vitro model of retinal neural cell death to investigate the neuroprotective ability of Müller glia derived EVs with a focus on RGC cells. Our results demonstrated that human Müller glia derived EVs aided cell survival by promoting neurite outgrowth of RGC-enriched cultures after NMDA-induced damage. Müller derived EVs were shown to modulate the activation of several phospho-kinases, which indicates the activation of pathways that are involved in neurite growth. The results suggest that Müller glia derived EVs may potentially constitute a therapeutic agent for retinal degenerative conditions.

Enrichment of RGCs cell cultures is important for modelling retinal diseases such as glaucoma as well as for the investigation of novel therapies. Traditionally, RGCs have been isolated from rodent retinae using immunopanning or other cell sorting methods, which constitute timely and costly procedures^20^. With the progress of stem cell organoid differentiation methods, there has been a drive towards generating differentiated RGCs of human origin, which is better matched for studying human diseases whilst reducing the need for using animal models. By replicating the pathways involved in human development, RGCs have been derived from human pluripotent stem cells. One such study uses a combination of noggin, DKK1, IGF1, N2 and B27 at various timings to induce embryoid body formation and then drive a retinal ganglion like neuronal cell fate^21^. Further enrichment via MACs cell sorting for Thy1.1 is then subsequently used to enrich the RGC like cultures^21^. Other labs have developed a BRN3B: mCherry reporter stem cell line to characterise the development of RGCs during retinal organoid formation. Cells are then dissociated and RGC like cells separated for further culture^22^. Although these studies prove effective in producing human RGCs for in vitro investigations, they require time consuming methods that may not be accessible to all laboratories. We have therefore sought to derive a simplified protocol of isolating human retinal neurons with an enriched population of RGC like cells without the need for cell sorting or reporter lines. Our study has shown effective isolation yield of 30–40% RGC-like cell population from 40 to 50 day old organoids derived from stem cells, which are plated onto laminin-coated tissue culture plates in the presence of a simple media cocktail consisting of DMEM F12, N2 supplement, NEAA and heparin. After 3–4 days in culture we show small neuronal like clusters that express characteristic markers of RGCs, including βIII tubulin, BRN3a/b, Thy1, γ-synuclein, RBMPS as well as NMDAR1^23–25^. We acknowledge that this cell preparation is not purely RGCs, but may contain other neuronal cell types. It is unlikely that any photoreceptors would be present in these populations due to the early age of the retinal organoids^13^, however isolated clusters may be made up of other retinal neuronal subtypes such as amacrine, bi-polar, horizontal cells, and retinal neuronal precursors that warrants further investigations. Despite these cell preparations containing a ‘mixed’ neuronal culture, we have shown that they contain a high proportion of RGC-like cells. Furthermore, as cells are isolated from human retinal organoids, other neuronal cell types will be of retinal origin and may still provide us with a tool to examine the ability of Müller glia for neuroprotection.

In the current study we show that Müller derived EV improved neurite outgrowth in isolated RGC-enriched clusters after NMDA induced damage. This EV population exerted a similar effect on the RGC-enriched cultures to that of the positive control, which consisted of neurotrophins BDNF, CNTF and forskolin. The use of these neurotrophins is well known to promote survival of RGCs in vivo and in vitro^26–29^. Müller glia are known to exert critical functions in metabolic, homeostatic and structural support of the retina^1,30^, and play an important role in promoting the survival of retinal neurons^31^. Our previous studies have demonstrated that transplantation of human Müller glia to RGC depleted rodent retinas can partially improve retinal function^5–7^. Despite this improvement, there is no evidence of integration of the cells, leading to the suggestion that Müller glia are releasing important trophic factors. Studies have shown that Müller glia can secrete important neurotrophic factors including BDNF, CNTF, bFGF, PEDF and GDNF^32–35^, which are known to improve RGC survivability in vitro^28,29,36^. The secretion of neurotrophic factors alone however, may not account for all the protective ability of Müller glia. Our recent studies have indicated that EVs released from Müller glia may provide some of this therapeutic effect. From our previous research we have identified multiple enriched miRNA and proteins that are within the Müller EV population and our preliminary investigations suggest downstream effects on cell proliferation, survival and growth^12^. Some of the most abundant miRNAs identified from the Müller cell populations include miR-21-5p, miR-16-5p, let-7a-5p, and miR-221-3p. Many studies have explored the roles MiR-21 in the central nervous system (CNS) and its potential not only as a biomarker but as a therapeutic target in CNS disorders. There is evidence for direct effects on anti-inflammatory signalling by targeting PDCD4 and promoting the anti-inflammatory cytokine IL-10^37^ and anti-apoptotic pathways through the regulation of AKT/PTEN/mTOR signalling axis in cortical neurons in vitro and in vivo^38,39^. MiR-16-5p and let-7a-5p have been identified as tumour suppressors in multiple cancer types^40–43^. Furthermore, both Let-7a-5p and miR-221-3p have also been associated with the promotion of axonal regeneration in rodent models of spinal cord injury via HMGA2/SMAD2 and p27 regulation respectively^44,45^. Due to the multiple identified roles of our most abundant Müller miRNAs, we sought to examine what effects the transfer of exosome content has on intercellular communication in our isolated RGC type cells by the investigation of the phosphorylation of multiple kinases.

Addition of NMDA for 24 h to the RGC-enriched cultures, followed by 24 h vehicle treatment showed an increase in activation of Mitogen-activated protein kinases (MAPKs) including, ERK1/2 (T202/Y204, T185/Y187), JNK1/2/3 (T183/Y185, T221/Y223), and P38α (T180/182). MAPKs act to direct cellular responses to various environmental stimuli that help direct cellular proliferation, differentiation, and apoptosis^46^. Similarly to other MAPKs, ERK1/2 signalling plays a role in cell proliferation as well as cell cycle arrest depending on the type of stimuli^47^. Although largely considered a pro-survival factor, studies have shown that persistent activation can be associated with glutamate-induced toxicity in primary cortical neuronal cells and that inhibition of ERK can protect against this toxicity^48^. JNK1,2,3 and P38α can be activated by environmental stress and have been identified as key regulators of apoptosis in neuronal cultures. In models of mouse optic nerve crush, JNK signalling is activated in RGC axons, where knockouts of *jnk2* and *jnk3* promoted RGC survival^49^. Similarly, studies have shown activation of JNK and p38α increases after induction of rodent retinal ischemia, and could be located to the nuclei of ganglion cells as well as the outer plexiform layer, and the nerve fiber layer^50^. In addition, blockade of p38 or ERK signalling provided some protection against this ischemic damage^50^, suggesting a role for inhibition of MAPK signalling in neuroprotection. Upregulation of these MAPKs, along with P53 in response to NMDA to the cell culture media in our current study suggests a reaction of the cells to environmental stress that may allude to the degenerative effect of NMDA on the RGC-enriched cultures. Furthermore, evidence that these pathways were then not significantly activated when EVs were applied after NMDA-induced damaged as compared to untreated controls, suggest that our Müller EVs may be providing some degree of neuroprotection. Interestingly we identified several kinases that were upregulated in response to the addition of EVs to the NMDA-damaged cells. These included RSK1/2 (S221/S227), RSK1/2/3 (S380/S386/S377), and p70 S6 kinase. The RSK family of kinases are downstream of effectors of the MAPK pathway and studies have shown that they have a prominent role in neuronal development and neuroprotection in the adult central nervous system (CNS). Examination of the role of RSK signalling in developing primary cortical neurons showed that inhibition significantly increased cell death, whilst assessment of mature hippocampal cultures showed that inhibition of RSK increased NMDA-induced cell death^51^. Consistent with this study, many others have reported suppression of neurite growth with addition of RSK inhibitors^52,53^, which suggests a role for RSK signalling in neuroprotection. Whilst these preliminary results suggest some potential mechanisms of EV signalling, these results generated by the phospho-kinase array are exploratory and semi-quantitative and have not been validated by the current study. As such this would warrant further investigations to confirm and elucidate the roles of EV in these cells.

Although EVs from multiple cell sources have been previously shown to be neuroprotective in retinal degeneration models, the effects of Müller derived EVs have not been extensively explored. As Müller glia are known to support normal retinal functioning, it may be suggested that their EV population would be more beneficial to the retinal environment than that of mesenchymal stem cells or other cell sources currently being investigated, that are not retinal cells. More work needs to be done to define the miRNA and protein populations contained in Müller glia EV that may exert the therapeutic effect, however results presented in this study provide a preliminary evidence for effect and suggest that Müller derived EVs may directly have a neuroprotective effect on human retinal neurons including RGCs and that they may be potentially used to develop treatments for retinal degenerative diseases such as glaucoma.

## Supplementary Information

Below is the link to the electronic supplementary material.

Supplementary Material 1

Supplementary Material 2

Supplementary Material 3

Supplementary Material 4

Supplementary Material 5

## Data Availability

All data generated or analysed during this study are included in this published article. If any further information is required the corresponding author may be contacted on reasonable request.
