# Supplementary material for: Müller glia derived EVs promote neurite recovery of an enriched population of retinal ganglion like cells derived from hESC retinal organoids after damage
Source: Sci Rep. 2026 Mar 3;16:11853. doi: 10.1038/s41598-026-42089-8 (PMC13065809; doi:10.1038/s41598-026-42089-8)
Supplement: Supplementary file 1 — Supplementary Material 1 [file 41598_2026_42089_MOESM1_ESM.pdf]

Supplementary Figure 1: Secondary antibody only controls for immunofluorescence staining.

Immunofluorescent images show secondary only controls performed on neural retinal cells isolated from hESC retinal organoids. (A) Images show cell nuclei DAPI (blue), and negative staining for donkey ant-goat 647, anti-rabbit 555 and anti-mouse 488 in combination. (B) Images show cell nuclei DAPI (blue), and negative staining for donkey ant-goat 647, anti-rabbit 488 and anti-mouse 555 in combination (C) Images show DAPI (blue) and negative staining for steptavivin dylight 549. Secondary antibodies all used at 1:500. Scale bars 50µm.

(A)

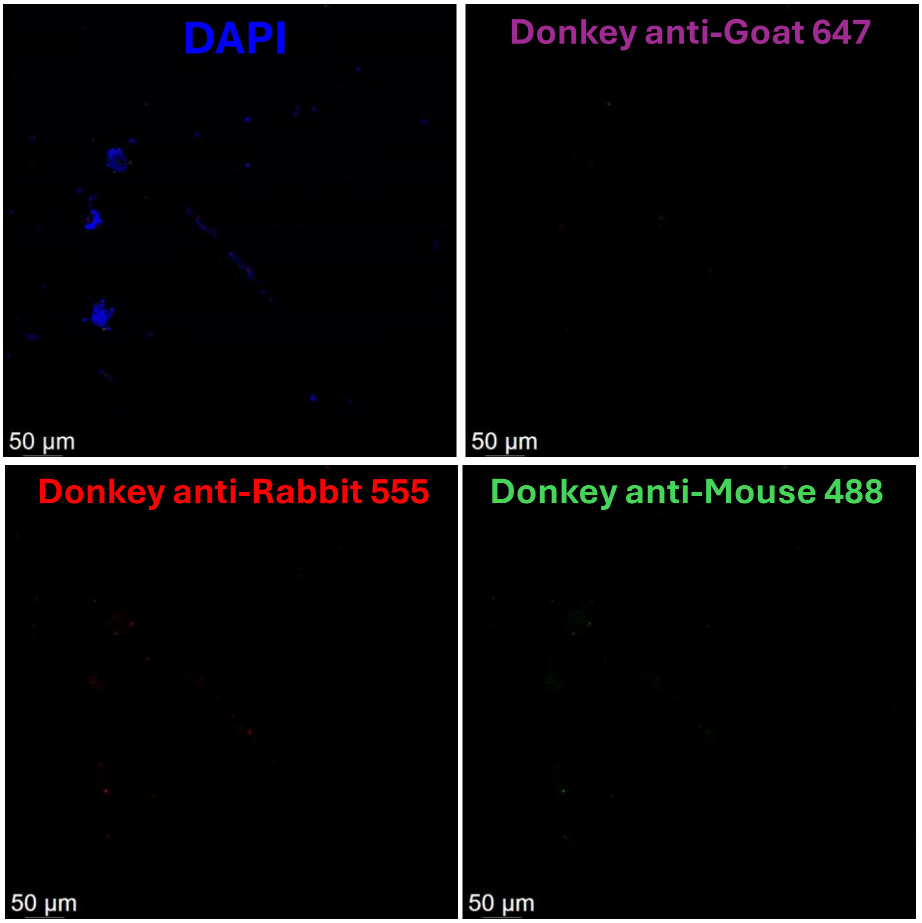

(B)

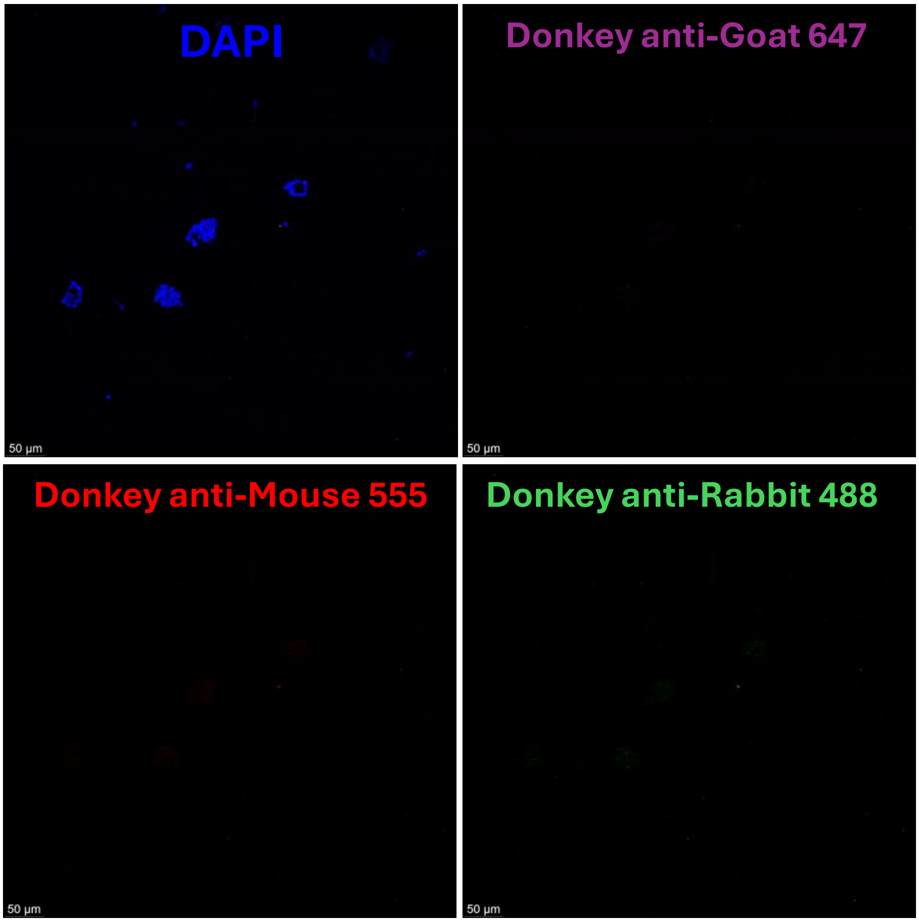
DAPI
50 µm
Donkey anti-Goat 647
50 µm
Donkey anti-Mouse 555
50 µm
Donkey anti-Rabbit 488
50 µm

(C)

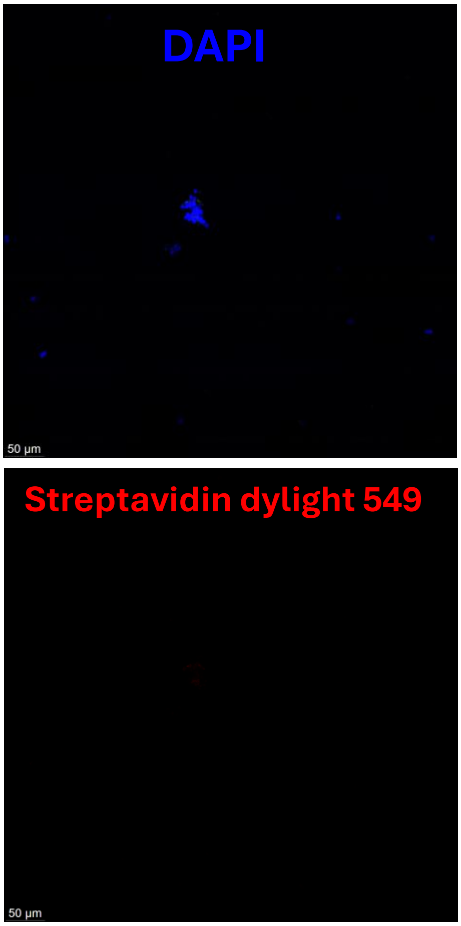
DAPI
50 µm
Streptavidin dylight 549
50 µm
