# Supplementary material for: Müller glia derived EVs promote neurite recovery of an enriched population of retinal ganglion like cells derived from hESC retinal organoids after damage
Source: Sci Rep. 2026 Mar 3;16:11853. doi: 10.1038/s41598-026-42089-8 (PMC13065809; doi:10.1038/s41598-026-42089-8)
Supplement: Supplementary file 2 — Supplementary Material 2 [file 41598_2026_42089_MOESM2_ESM.pdf]

Supplementary Figure 2: Proteome profiler human phospho-kinase dot blot array.
(A,B,C,D) Images show the Raw chemiluminescent dot blot arrays showing levels of phospo-kinase proteins detected in untreated control RGC-enriched cultures (control), as compared to those treated with NMDA for 24hrs with or without Müller EV treatment for a further 24hrs (NMDA + vehicle, NMDA + EV). The four replicates are shown (A,B,C,D).
(E) Guide supplied by manufacturer used to identify proteins detected in dot bot array.

Chemiluminescent Dot blot array _ raw images

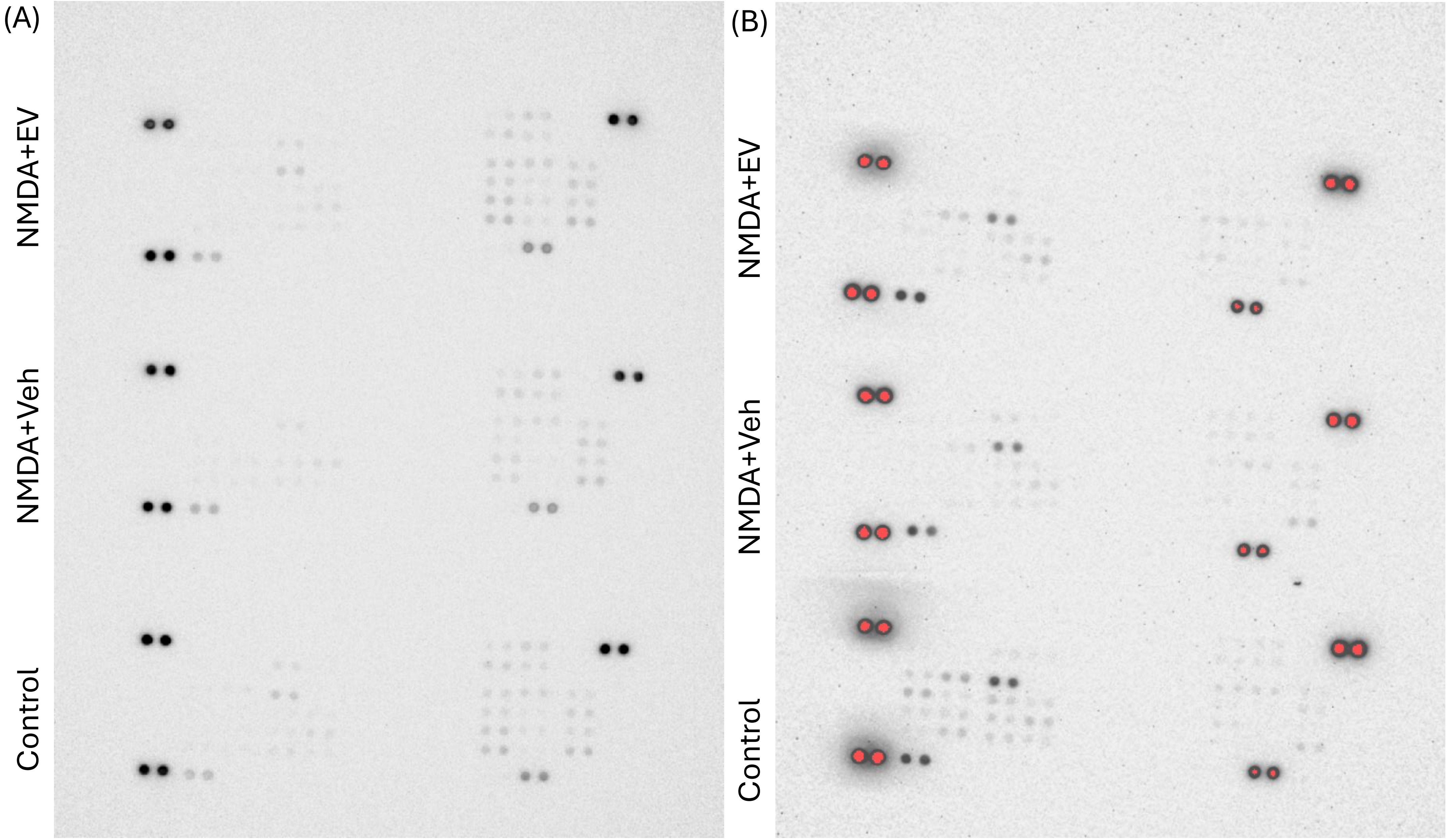

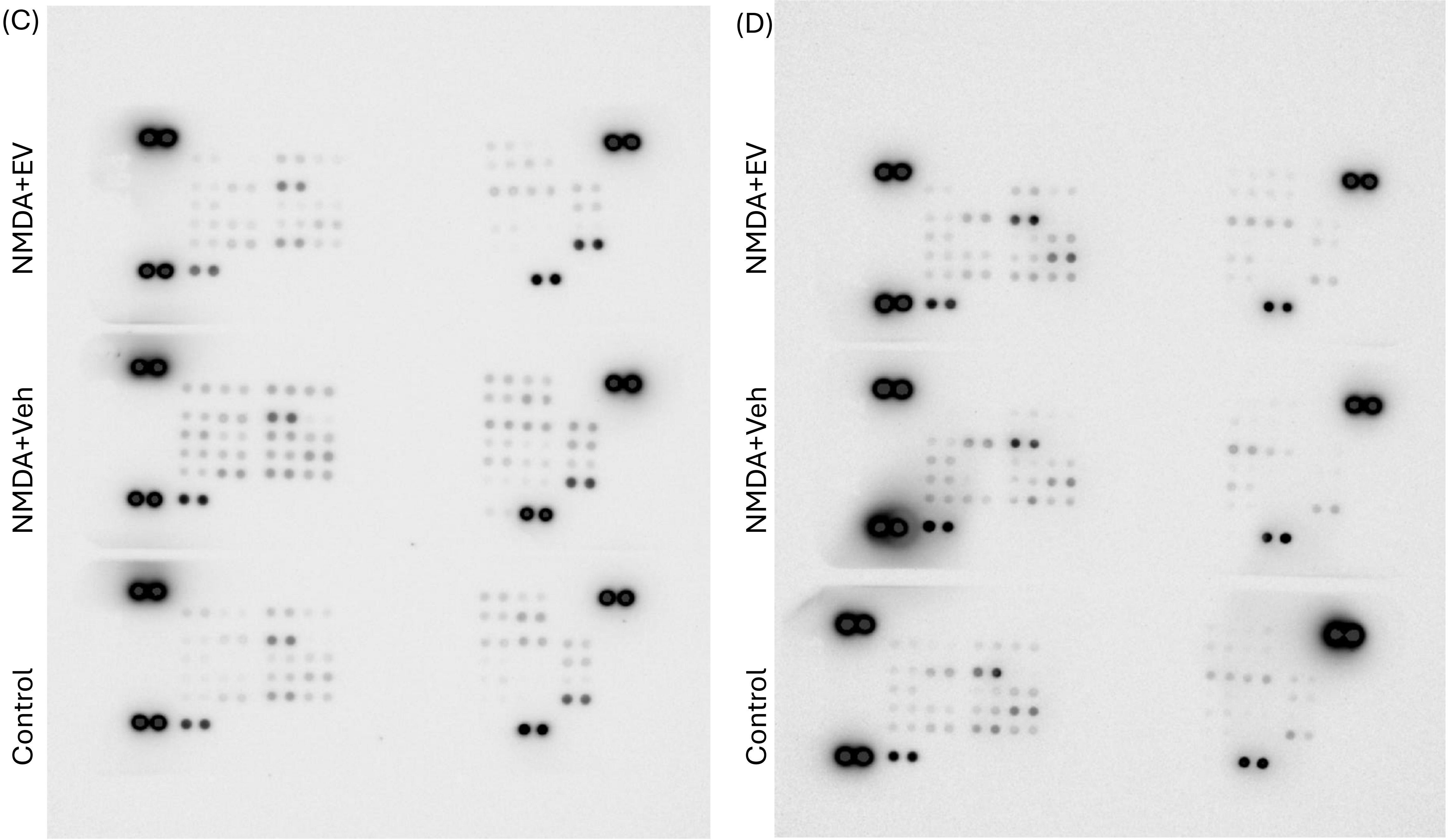
(C)
NMDA+EV
NMDA+Veh
Control
(D)
NMDA+EV
NMDA+Veh
Control

(E) Human Phospho-Kinase Array Coordinates

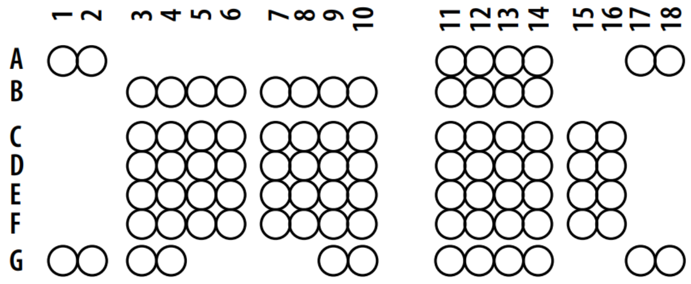

Membrane/ Coordinate	Target/Control	Phosphorylation Site
A-A1, A2	Reference Spot	——
B-A11, A12	Akt 1/2/3	T308
B-A13, A14	Akt 1/2/3	S473
B-A17, A18	Reference Spot	——
A-B3, B4	CREB	S133
A-B5, B6	EGF R	Y1086
A-B7, B8	eNOS	S1177
A-B9, B10	ERK1/2	T202/Y204, T185/Y187
B-B11, B12	Chk-2	T68
B-B13, B14	c-Jun	S63
A-C3, C4	Fgr	Y412
A-C5, C6	GSK-3α/β	S21/S9
A-C7, C8	GSK-3β	S9
A-C9, C10	HSP27	S78/S82
B-C11, C12	p53	S15
B-C13, C14	p53	S46
B-C15, C16	p53	S392
A-D3, D4	JNK 1/2/3	T183/Y185, T221/Y223
A-D5, D6	Lck	Y394
A-D7, D8	Lyn	Y397
A-D9, D10	MSK1/2	S376/S360
B-D11, D12	p70 S6 Kinase	T389
B-D13, D14	p70 S6 Kinase	T421/S424
B-D15, D16	PRAS40	T246

Membrane/ Coordinate	Target/Control	Phosphorylation Site
A-E3, E4	p38α	T180/Y182
A-E5, E6	PDGF Rβ	Y751
A-E7, E8	PLC-γ1	Y783
A-E9, E10	Src	Y419
B-E11, E12	PYK2	Y402
B-E13, E14	RSK1/2	S221/S227
B-E15, E16	RSK1/2/3	S380/S386/S377
A-F3, F4	STAT2	(pY690)
A-F5, F6	STAT5a/b	Y694/Y699
A-F7, F8	WNK1	T60
A-F9, F10	Yes	Y426
B-F11, F12	STAT1	Y701
B-F13, F14	STAT3	Y705
B-F15, F16	STAT3	S727
A-G1, G2	Reference Spot	——
A-G3, G4	β-Catenin	——
A-G9, G10	PBS (Negative Control)	——
B-G11, G12	STAT6	Y641
B-G13, G14	HSP60	——
B-G17, G18	PBS (Negative Control)	——
