# Supplementary material for: Müller glia derived EVs promote neurite recovery of an enriched population of retinal ganglion like cells derived from hESC retinal organoids after damage
Source: Sci Rep. 2026 Mar 3;16:11853. doi: 10.1038/s41598-026-42089-8 (PMC13065809; doi:10.1038/s41598-026-42089-8)
Supplement: Supplementary file 3 — Supplementary Material 3 [file 41598_2026_42089_MOESM3_ESM.pdf]

Supplementary Table 1. List of primary antibodies used in the study.

Table lists all primary antibodies used for immunofluorescence staining in the study, including the source and dilution used.

**Antibody**	**Host**	**Source**	**Dilution**
**βIII tubulin (TuJ1)**	Rabbit	Abcam (Ab18207)	1:1000
**βIII tubulin (TuJ1)**	Mouse	Biolegend (801201)	1:1000
**BRN3 (pan)**	Goat	Santa Cruz (SC6026)	1:50
**BRN3B**	Goat	Santa Cruz (SC31987)	1:50
**SOX2**	Goat	Santa Cruz (SC17319)	1:50
**γ-synuclein**	Mouse	Santa Cruz (SC65979)	1:100
**PAX6**	Rabbit	Abcam (Ab5790)	1:100
**THY1**	Mouse	Biolegend (328106)	1:100
**NMDAR1**	Rabbit	Abcam (Ab109182)	1:100
**RBPMS**	Mouse	Novus Biologicals (NBP2-03905)	1:50
