# Supplementary material for: Müller glia derived EVs promote neurite recovery of an enriched population of retinal ganglion like cells derived from hESC retinal organoids after damage
Source: Sci Rep. 2026 Mar 3;16:11853. doi: 10.1038/s41598-026-42089-8 (PMC13065809; doi:10.1038/s41598-026-42089-8)
Supplement: Supplementary file 4 — Supplementary Material 4 [file 41598_2026_42089_MOESM4_ESM.pdf]

Supplementary Table 2: Numbers of images, neurite clusters and cells used for neurite analyses.

Table shows the number of images analysed for each sample type, the average number of clusters per replicate and the average number of cells per cluster of neurites that were used for taking neurite measurements.

N=7	Ctrl	Ctrl + EV	NMDA	NMDA + EV	NMDA + NT	NMDA + MK
**Total No. of images analysed:**	26	16	25	21	14	12
**Average no. clusters analysed per replicate (mean ± SD)**	10 ± 2.5	10 ± 2.6	10 ± 2.2	12 ± 1.8	15 ± 3.0	11± 1.1
**Average no. of cells per cluster (mean ± SD)**	30.7 ± 23.3	33.9 ± 47.2	24.2 ± 21.0	32.2 ± 33.1.8	22.4 ± 14.2	28.3 ± 17.8
